# All-Solid-State Thin-Film Lithium-Sulfur Batteries

**DOI:** 10.1007/s40820-023-01064-y

**Published:** 2023-03-27

**Authors:** Renming Deng, Bingyuan Ke, Yonghui Xie, Shoulin Cheng, Congcong Zhang, Hong Zhang, Bingan Lu, Xinghui Wang

**Affiliations:** 1 College of Physics and Information Engineering, Institute of Micro-Nano Devices and Solar Cells, Fuzhou University, Fuzhou, 350108 People’s Republic of China; 2 Fujian Science & Technology Innovation Laboratory for Optoelectronic Information of China, Fuzhou, 350108 Fujian People’s Republic of China; 3 Jiangsu Collaborative Innovation Center of Photovoltaic Science and Engineering, Changzhou, 213000 People’s Republic of China; 4 School of Physics and Electronics, Hunan University, Changsha, 410082 People’s Republic of China

**Keywords:** All-solid-state thin-film batteries, Li-S batteries, Vertical graphene nanosheets, Lithium phosphorous oxynitride, Li_2_S

## Abstract

Lithium-sulfur (Li–S) system coupled with thin-film solid electrolyte as a novel high-energy micro-battery has enormous potential for complementing embedded energy harvesters to enable the autonomy of the Internet of Things microdevice. However, the volatility in high vacuum and intrinsic sluggish kinetics of S hinder researchers from empirically integrating it into all-solid-state thin-film batteries, leading to inexperience in fabricating all-solid-state thin-film Li–S batteries (TFLSBs). Herein, for the first time, TFLSBs have been successfully constructed by stacking vertical graphene nanosheets-Li_2_S (VGs-Li_2_S) composite thin-film cathode, lithium-phosphorous-oxynitride (LiPON) thin-film solid electrolyte, and Li metal anode. Fundamentally eliminating Li-polysulfide shuttle effect and maintaining a stable VGs-Li_2_S/LiPON interface upon prolonged cycles have been well identified by employing the solid-state Li–S system with an “unlimited Li” reservoir, which exhibits excellent long-term cycling stability with a capacity retention of 81% for 3,000 cycles, and an exceptional high temperature tolerance up to 60 °C. More impressively, VGs-Li_2_S-based TFLSBs with evaporated-Li thin-film anode also demonstrate outstanding cycling performance over 500 cycles with a high Coulombic efficiency of 99.71%. Collectively, this study presents a new development strategy for secure and high-performance rechargeable all-solid-state thin-film batteries.

**Supplementary Information:**

The online version contains supplementary material available at 10.1007/s40820-023-01064-y.

## Highlights

- The all-solid-state thin-film Li-S battery has been successfully developed by stacking VGs-Li_2_S cathode, lithium-phosphorous-oxynitride (LiPON) solid electrolyte, and Li anode.
- The obtained VGs-Li_2_S thin-film cathode exhibits excellent long-term cycling stability (more than 3,000 cycles), and an exceptional high temperature tolerance (up to 60 °C).
- The superb electrochemical performance can be attributed to the favorable compatibility and outstanding interfacial stability between VGs-Li_2_S thin film and LiPON.

**Supplementary Information:**

The online version contains supplementary material available at 10.1007/s40820-023-01064-y.

## Graphical abstract

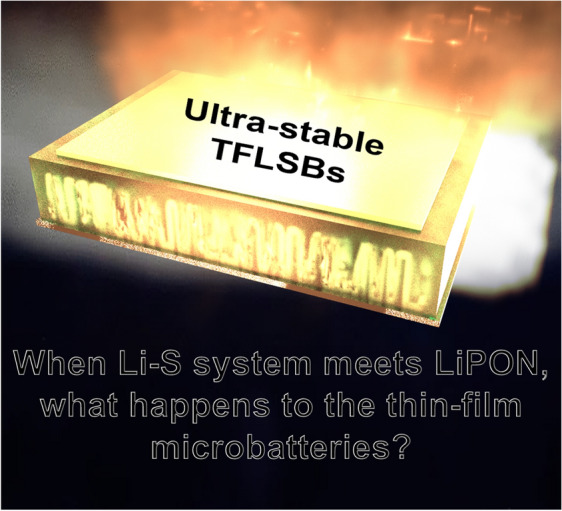

## Introduction

Practically implementing autonomy on the extreme edge nodes of the Internet of Things (IoT) requires a miniature energy storage device that features a small volume, light weight, high energy, and easy integratability for perpetual energy supply (over ten years) [1–3]. Considering thin-film architectures and layer-by-layer stacking fabrication strategy, all-solid-state thin-film batteries (TFBs) have become particularly attractive in powering IoT microdevices such as smart cards, microsensors, microelectronics, and micromechanical devices that could not be replaced with other type batteries, since their on-chip-integratable and shape-variable features can effectively utilize the residual spaces [4–8]. However, TFBs based on conventional cathode materials (such as intercalated lithium metal oxides, whose theoretical capacities are normally lower than 100 μAh cm^−2^ μm^−1^) exhibit inferior volumetric and areal energy densities, mismatching with those of high-capacity anodes, such as lithium (196.5 μAh cm^−2^ μm^−1^) and silicon (834 μAh cm^−2^ μm^−1^) [9–11]. Therefore, efforts for replacing conventional cathodes with high-energy–density materials are mandatory. Lithium-sulfur (Li–S) systems have been widely used in liquid-electrolyte systems due to the outstanding theoretical capacity of S cathode (1675 mAh g^−1^) and remarkably high-energy-density (2,600 Wh Kg^−1^), accompanied by the notorious drawback of the shuttle effect and huge volume changes during cycling [12–17]. Many strategies have been devoted to tackle these challenges including designing novel cathodes, modifying separators, and replacing liquid electrolytes with solid-state electrolytes [18–22]. Among them, bulk-type solid-state Li-S batteries have recently received enormous attention due to completely inhibiting the dissolution of Li-polysulfides and fundamentally eliminating the shuttle effect [22–26].

In this regard, an intriguing research question stands out: Is it possible to transfer the Li-S system into the field of TFBs? It is believed that achieving high performance is particularly difficult for all-solid-state Li-S batteries whether in the fields of bulk or thin-film types, considering the intrinsic sluggish kinetics of S and Li_2_S [26, 27]. Also, notorious lithium dendrite growth as well as the correlative cathode/electrolyte or anode/electrolyte interface degradation issues have become a practical obstacle for the application of Li-S systems into solid-state fields [28–32]. Substantial efforts have been devoted to improving the interfacial charge transfer kinetics by introducing large amounts of carbon and solid electrolyte [33–35]. However, a TFB fabricated with a thin-film-stacked configuration is difficult to direct integration of the downscaled powder-based cathodes owing to the insufficient flatness, uncontrollable thickness, and unsophisticated processing technic, which gives rise to the deposition defect of lithium phosphorous oxynitride (LiPON), inadequate energy utilization, and going against with the integration principle of TFBs [24, 36]. Hence, the cathode for TFBs principally needs to be designed as flat thin-film form and the overall thickness of battery is usually lower than 20 μm [37]. In addition, the volatility of S typically leads to the loss of active materials and significant contamination of equipment during the sputtering process at an elevated vacuum [23, 38].

Based on this, S has been replaced with fully lithiated Li_2_S in this work for a higher melting point of 938 ℃ and lower density (1.66 g cm^−3^), which can be stabilized during the subsequent sputtering process at high vacuum and minimize the volume expansion upon charge/discharge cycling since Li_2_S is already at the maximum volume [39–41]. The vertical graphene nanosheets (VGs) with excellent electronic conductivity, large specific surface area, and inherent reticular structure are employed as the three-dimensional conductive host for Li_2_S, which are expected to not only effectively relieve the stress/strain of active materials, but also build the favorable conductive channels and promote the electrochemical stability of the active materials [20, 42, 43].

A primary consideration in the successful fabrication of an all-solid-state thin-film Li-S battery (TFLSB) is pairing it with a compatible electrolyte. LiPON as solid electrolyte is an important part of TFBs due to its negligible electronic conductivity, appropriate ionic conductivity (2.3 ± 0.7 × 10^−6^ S cm^−1^), low activation energy (0.55 eV), and wide electrochemical stability window (0–5.5 V vs. Li/Li^+^) [44–47]. As a successfully commercialized thin-film solid electrolyte, LiPON is demonstrated to be electrochemically stable against a variety of sulfide materials [48–52]. The conversion sulfide cathodes experience lithiated reactions into nano-metal and Li_2_S, which implies the feasibility of integrating Li_2_S cathode into TFBs [53]. What’s more, the inherent wide electrochemical stability window and the ability to suppress Li penetration make LiPON well accommodated with Li anode [54, 55]. Therefore, it’s expected that the LiPON-based thin-film solid electrolyte can stabilize both the cathode and anode interface and Li-S systems can be well transferred into the untrodden zone of TFBs.

In this work, for the first time, TFLSBs were successfully created by stacking VGs-Li_2_S composite thin-film cathode, LiPON thin-film solid electrolyte, and Li anode. Full cells exhibited superior electrochemical stabilization, demonstrating the feasibility of transferring Li-S systems into TFBs. The excellent electrochemical durability of VGs-Li_2_S composite thin-film cathode for 3,000 cycles is attributed to the favorable compatibility and outstanding interfacial stability between thin-film solid electrolyte with VGs-Li_2_S composite thin film, which radically suppresses the “shuttle effect”. Furthermore, the developed solid-state Li-S system could work at high temperature up to 60 °C with a high areal capacity of 20.47 μAh cm^−2^. More importantly, VGs-Li_2_S-based TFLSBs could deliver a discharge capacity of 5.52 μAh cm^−2^ for 500 cycles at 10 μA cm^−2^. To our knowledge, this is the first time to demonstrate the cyclability of VGs-Li_2_S-based TFLSBs (VGs-Li_2_S/LiPON/Li), which shows tremendous potential for further improving the areal and volumetric energy densities of TFBs. These results laid a foundation for Li_2_S-based cathode material in TFBs, which could provide guidance in designing next-generation high-energy-density TFLSBs.

## Experimental Section

### Preparation of VGs

VGs were prepared by a previously reported method via the plasma-enhanced chemical vapor deposition (PECVD) route (BEQ BTF-1200C-S-SL-PECVD) [20]. A stainless steel (SS) substrate was selected for deposition in a PECVD chamber. The reaction temperature and time were 600 °C and 60 min, respectively. The power of radio-frequency plasma was 80 W.

### Preparation of VGs-Li_2_S Thin-film Cathode

Electrodeposition of Li_2_S was performed with Li foil as counter/reference electrode and VGs coated SS as working electrode. 30 μL of the Li-S electrolyte (1.0 M lithium bis(trifluoromethanesulfonyl)imide (LiTFSI) in a binary solvent mixture of 1,3-dioxolane (DOL) and dimethoxymethane (DME) (1:1 in volume) with 1 wt% LiNO_3_ as the additive) and 10 μL of Li_2_S_6_ catholyte (0.6 M) were used as the electrodeposition electrolyte. The 0.6 M Li_2_S_6_ catholyte was prepared by dissolving S and Li_2_S in a molar ratio of 5:1 into the above Li–S electrolyte under magnetic stirring at 80 °C for 48 h. The assembled coin cells were charged and discharged in galvanostatic mode using a Neware BTS-4000 multichannel battery tester. To make the deposition process of Li_2_S more complete, after shelving for 6 h, the coin cell was cycled at 1–3 V with a constant current of 100 μA and then deep discharge at the cut-off voltage for about 10 h. Then the VGs-Li_2_S electrode after electrodeposition was rinsed with DOL solution and heated at 250 °C for 3 h. All the processes were done in a glove box filled with argon (H_2_O < 0.1 ppm, O_2_ < 0.1 ppm).

### Preparation of VGs-Li_2_S/LiPON/Li Cell

First, 1.5 μm LiPON was deposited on VGs-Li_2_S thin film cathode at room temperature (RT) by Radio Frequency magnetron sputtering (BEIJING TECHNOL CO., LTD) with a stoichiometric Li_3_PO_4_ target at a pressure of 0.6 Pa, N_2_ gas flow of 30 sccm and a power of 90 W. After that, the anode was fabricated by pressing Li (Pre-Li) foil or evaporated-Li (Evp-Li) thin film on the surface of LiPON. The area of Pre-Li is 0.190 cm^2^ and the Evp-Li is 0.196 cm^2^. The final fabrication step of the VGs-Li_2_S/LiPON/Evp-Li is the deposition of the Ti current collector on top. The Ti thin-film was selected as the current collector for its good electrical conductivity, electrical conductivity, and high oxidation resistance. Besides, VGs-Li_2_S thin-film cathode-based liquid Li-S batteries were also assembled by using Li-S electrolyte and a Li foil as anode for performance comparison.

### Material Characterization

The morphological analyses and elemental mapping distribution were performed using an FEI Helios G4-CX SEM and the accompanying EDS system. The morphology and structure were further characterized by JEOL JEM-2800 TEM. AFM analyses (5500, Agilent) were conducted over an area of 4 μm × 4 μm.

### Electrochemical Measurements

The VGs-Li_2_S/LiPON/Pre-Li and VGs-Li_2_S/LiPON/Evp-Li cells were both assembled in an argon-filled glove box using CR2025-type coin cell as a sealing case without liquid electrolyte. Galvanostatic charge and discharge measurements were conducted using a Neware multichannel battery testing system (BST-4000) with a voltage window of 1.0–4.0 V. Cyclic voltammetry (CV) and Electrochemical impedance analyses (EIS) tests were performed on an SP-200 Biologic electrochemical workstation.

## Results and Discussion

### Preparation and Characterization of VGs-Li_2_S Thin Film

**Fig. 1:**
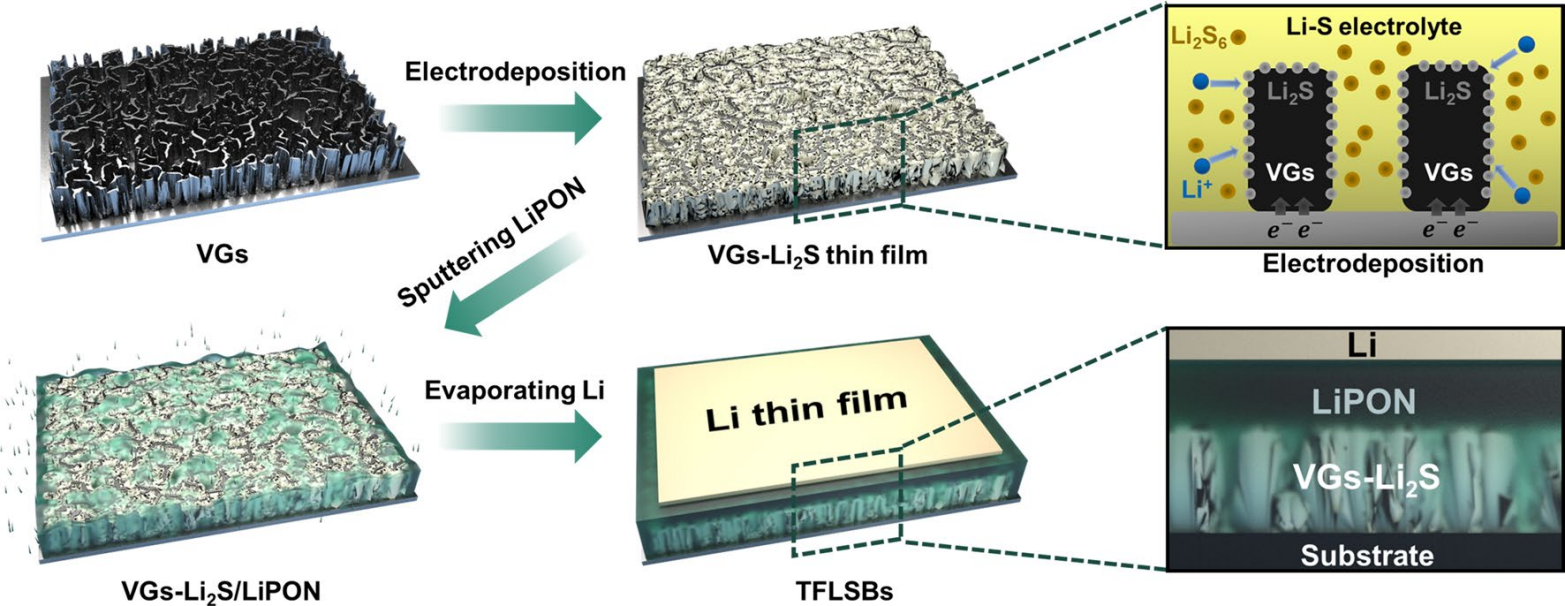
Schematic illustration of the fabricated TFLSBs

The schematic illustration of the VGs-Li_2_S-based TFLSB fabrication procedures is presented in Fig. 1. The VGs-Li_2_S composite thin film with a thickness of 600 nm was constructed by the electrodeposition of Li_2_S into VGs current collector. To visually analyze the electrochemical reactions involved in this study, the associated voltage profiles are plotted in Fig. S1. The first charging process is related to oxidizing Li_2_S_6_ to S_8_, and subsequent discharging process displays two characteristic discharge plateaus at 2.3 and 2.1 V, implying S_8_ is reduced stepwise, undergoing high-order polysulfides (Li_2_S_x_, 6 < X ≤ 8), low-order polysulfides (Li_2_S_x_, 2 < X ≤ 6) and lithium disulfide (Li_2_S_2_), to finally lithium sulfide (Li_2_S) [56, 57].

**Fig. 2:**
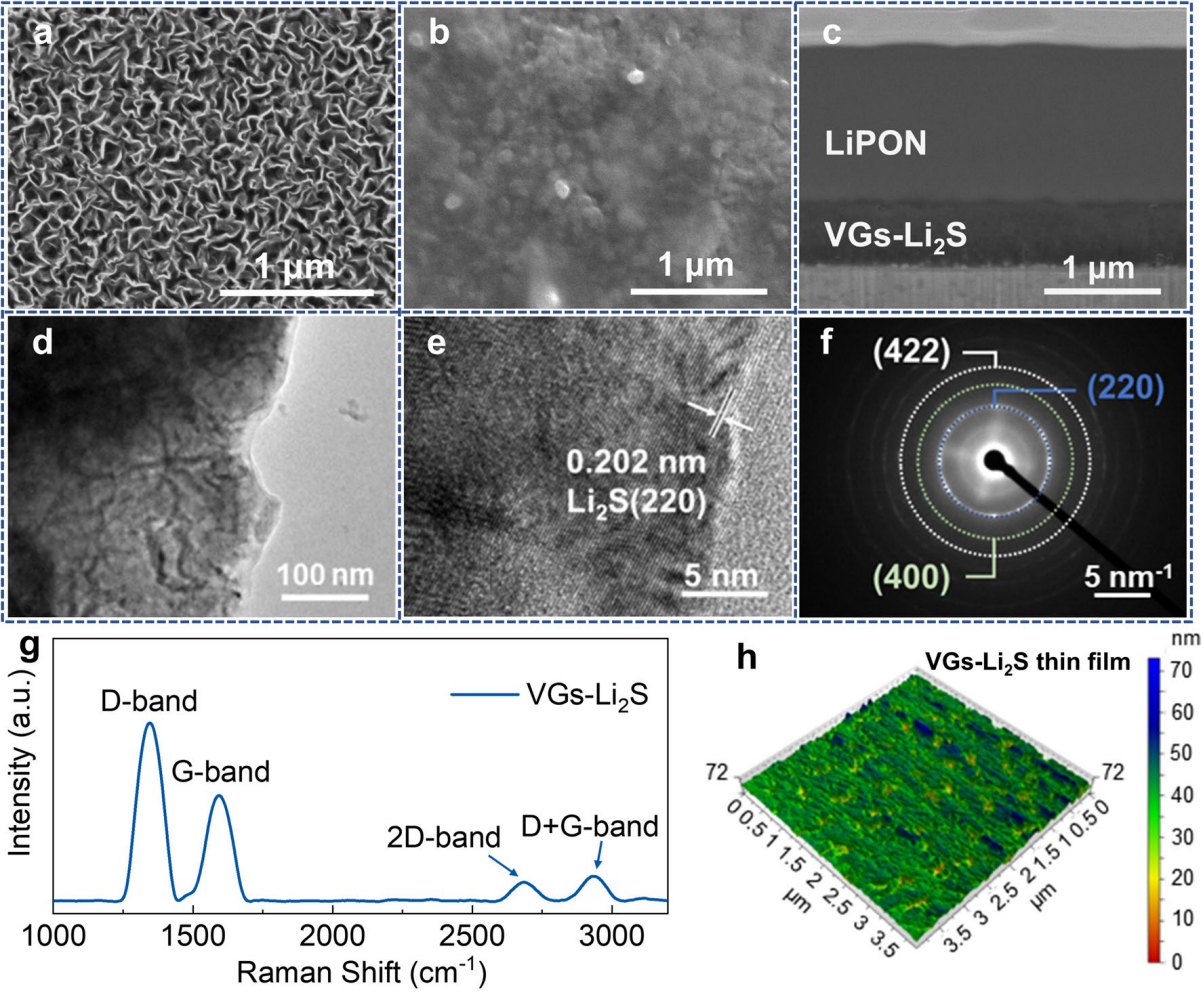
The surface SEM morphology of **a** VGs and, **b** VGs-Li_2_S composite thin film. **c** FIB-SEM images of the VGs-Li_2_S/LiPON interface. **d** TEM image, **e** HRTEM image, **f** SAED pattern, **g** Raman spectra, and **h** AFM topographic images of VGs-Li_2_S thin film

The scanning electron microscope (SEM) morphology characterization of cathode thin film before and after electrodeposition Li_2_S is observed in Fig. 2a, b, respectively. Figure 2a shows the VGs sample have a uniform, ordered, and interconnected sheet structure, which could provide an ideal conductive environment in vertical space for Li_2_S electrodeposition (Fig. S2). After electrodeposition, it can be seen that the VGs are uniformly covered by membraniform Li_2_S (Fig. 2b). The cross-sectional SEM image (Fig. 2c) reveals that the VGs-Li_2_S/LiPON has intimate solid–solid contact interface, which is well-defined without any defects reflecting the risk of battery failure. Additionally, the thickness of the VGs-Li_2_S composite film is about 700 nm and the Li_2_S fills the void space in between VGs effectively.

Spatially resolved characterization of the VGs-Li_2_S composite materials with a scale bar of 100 and 5 nm was investigated by transmission electron microscopy (TEM) and high-resolution TEM (HRTEM), respectively. Figure 2d shows a typical interconnected sheet-like network, consistent with the SEM results. HRTEM image of the sample (Fig. 2e) reveals that the crystal fringe is 0.202 nm, which can be assigned to the (220) lattice plane of cubic Li_2_S (JCPDS No. 77–2145). In addition, the selected area electron diffraction (SAED) pattern (Fig. 2f) and fast Fourier transform (FFT) results (Fig. S3) confirm the high crystallinity of the Li_2_S components. Figure S4 displays a lattice spacing of approximate 0.35 nm, which can be attributed to the (002) lattice plane of few-layer graphene. Figure 2g shows the Raman spectrum of VGs-Li_2_S measured by a laser with a wavelength of 532 nm. Four peaks are seen in the spectrum located at 1350, 1590, 2680, and 2930 cm^−1^, corresponding to the typical characteristic D, G, 2D, and D + G peaks of VGs, respectively [43]. However, there are no characteristic peaks of Li_2_S observed in this spectrum because the signal of Li_2_S is too weak compared to VGs. The surface roughness of the VGs-Li_2_S thin film on the SS substrate is shown in three-dimensional atomic force microscopy (AFM) images (Fig. 2h). The root-mean square height (Sq) of the thin film sample in 4 μm × 4 μm is 6.75 nm, guaranteeing the sufficient flatness to avoid functionality failure of LiPON during cycling.

### Electrochemical Performance of VGs-Li_2_S Thin-Film Cathode

**Fig. 3:**
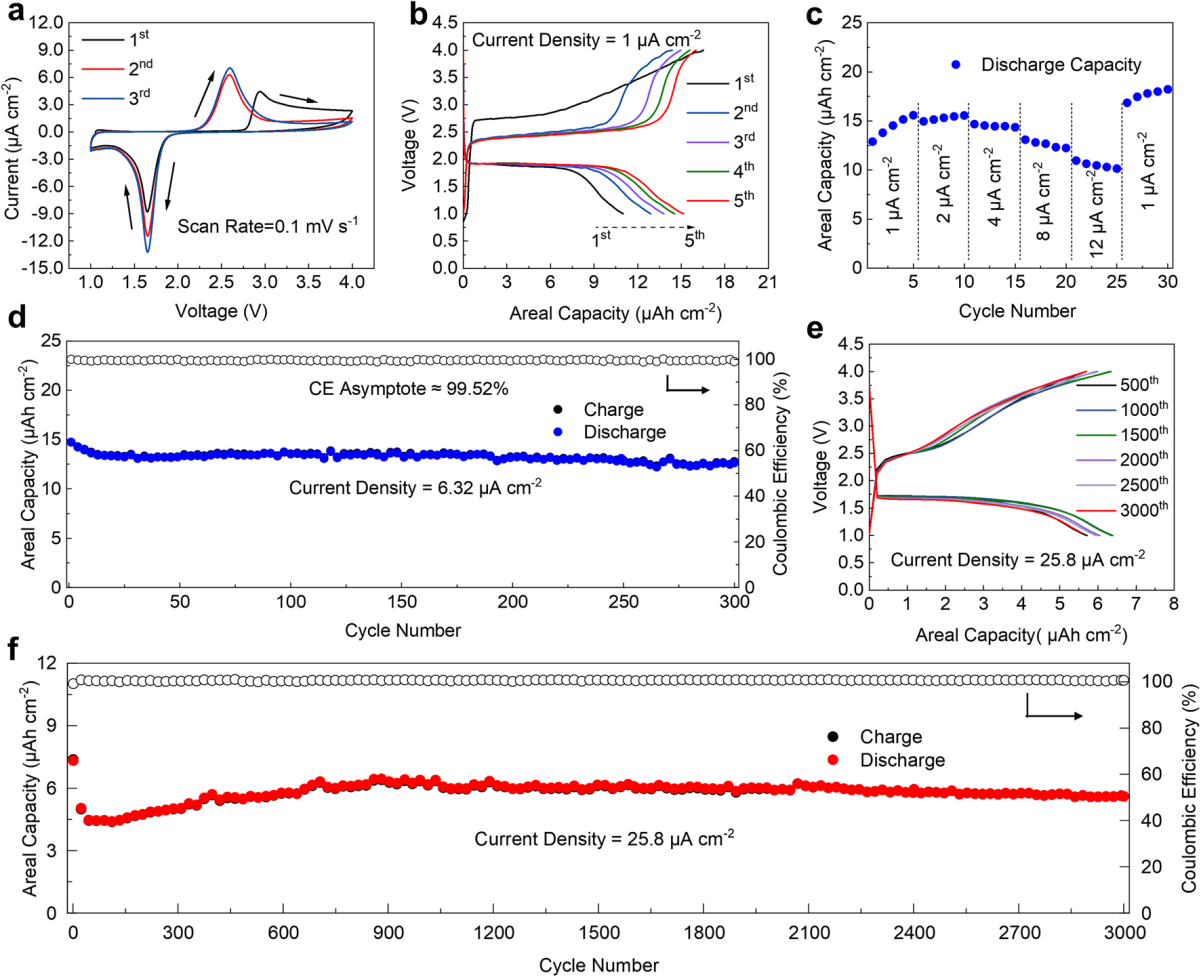
Electrochemical performance of VGs-Li_2_S/LiPON/Pre-Li cells. **a** CV curves at a scan rate of 0.1 mV s^−1^, **b** initial five voltage profiles at a current density of 1 μA cm^−2^, **c** rate performance, **d** cycling performances at 6.32 μA cm^−2^, **e** voltage profiles at different cycles, and **f** long-term cycling performance at 25.8 μA cm^−2^

To evaluate the electrochemical performance of VGs-Li_2_S cathode in solid-state systems, the model configuration (VGs-Li_2_S/LiPON/Pre-Li) was employed by stacking VGs-Li_2_S thin-film cathode, LiPON solid electrolyte, and Li foil as an “unlimited Li” reservoir. Figure 3a shows the CV of VGs-Li_2_S/LiPON/Pre-Li cells in the range of 1–4 V. In the initial anodic scan, the anodic peak at 2.95 V can be attributed to the oxidation of Li_2_S to S. Such an elevated oxidation peak is a universal initial energy/voltage barrier for Li–S batteries using Li_2_S-based cathode, illustrating the cell generally require higher activation voltage to extracted Li from Li_2_S [58–60]. A couple of redox peaks at 1.65 and 2.6 V are observed in the following CV scan, corresponding to the one-step redox reaction between S_8_ and Li_2_S with the absence of the Li-polysulfide intermediates [23, 24]. In contrast, the CV curves of the VGs-Li_2_S thin-film cathode in liquid Li-S system (Fig. S5a) exhibit two pair of redox peaks due to the two-step redox reaction, which are consistent with many reported results [20, 61, 62]. Therefore, compared with the classic conversion processes in liquid Li-S systems, solid-state configuration confirms the disappearance of Li-polysulfides upon cycling, inhibiting the shuttle effect. In the subsequent cycling, redox peak intensities increase gradually due to the activation of extra Li_2_S [63].

Figure 3b shows the voltage profiles of the VGs-Li_2_S cathode at the first 5 cycles under 1 μA cm^−2^. It is consistent with CV curves for Li_2_S to present an initial charge plateau at 2.8 V that originated from the overlarge delithiated energy barrier in solid-state systems. There is only one plateau in the subsequent charge/discharge processes, which are agreed well with the typical solid-to-solid binary phase transition (between Li_2_S and S_8_) behavior and differed with the liquid Li-S battery that has two plateaus (Fig. S5b). The VGs-Li_2_S cathode could deliver an initial charge and discharge capacity of 16.52 and 10.99 μAh cm^−2^ respectively, corresponding to a low initial Coulombic Efficiency (CE) of 66.5%, which might originate from the imperfect solid–solid contact and insufficient ionic/electronic conductivity after charging [40, 41, 59]. Significantly, the charge/discharge capacity gradually increases along with the cycling, because Li_2_S was continuously activated as the cycling progresses.

In rate studies (Fig. 3c), the VGs-Li_2_S thin-film cathode displayed the reversible capacity of 15.58, 14.95, 14.68, 13.11, and 10.95 μAh cm^−2^ with the current density increasing from 1 to 2, 4, 8, and 12 μA cm^−2^, respectively. Furthermore, the capacity even reversibly recovers to a value higher than the initial low-rate cycling once the current density goes back to 1 μA cm^−2^. In addition, the corresponding charge/discharge voltage profiles at various current densities are shown in Fig. S6, indicating that there are obvious flat charge/discharge plateaus even at high rates. It is clear that the polarization just only slightly increases with increasing charge/discharge current densities, which redounds to its high-rate performance. The excellent rate performance can be attributed to the intrinsic three-dimensional conductive networks of VGs along with intimate solid–solid contact between LiPON thin film and VGs-Li_2_S cathode. The galvanostatic cycling performance of the VGs-Li_2_S thin-film cathode was evaluated at 6.32 μA cm^−2^. As illustrated in Fig. 3d, the VGs-Li_2_S cathode displays excellent cycling stability with a high-capacity retention of 98.8% from 20 to 300 cycles by obtaining a 99.52% CE averaged over every cycle.

Furthermore, long-term cycling performance of the VGs-Li_2_S thin-film cathode was conducted at a higher current density of 25.8 μA cm^−2^ after a smaller current activation (Fig. S7). Figure 3e shows the galvanostatic charge/discharge voltage profiles of the VGs-Li_2_S thin-film cathode at different cycles. The well-overlapped curves are observed for every 500 cycles suggested the favorable compatibility of thin-film solid electrolyte with VGs-Li_2_S composite thin film preliminarily. Having a closer look at Fig. 3f, the discharge capacity reduced to 4.55 μAh cm^−2^ after 80 cycles, which originated from the incomplete redistribution of the sheet-like Li_2_S over the VGs networks during the initial high current activation. Subsequently, the discharge capacity gradually increased up to a maximum value of 6.7 μAh cm^−2^ associated with a gradual activation process of Li_2_S. The discharge capacity of 5.79 μAh cm^−2^ was maintained over 3,000 cycles, which firmly demonstrates the superiority of the VGs-Li_2_S thin film cathode for integrating into the LiPON solid-state system and maintaining the cycling stabilities in compared with the liquid systems (Fig. S5c). This remarkable cycle performance of the VGs-Li_2_S thin-film cathode is also believed to be attributed to the effective inhibition of polysulfide shuttle behavior because of the otherness of ion transport in solid-state systems [23]. It is worth to mention that the VGs-Li_2_S/LiPON/Pre-Li cell could drive a thermometer with the temperature of the fingertips at 35.7 °C (Fig. S8), demonstrating the great potential for micro-device.

**Fig. 4:**
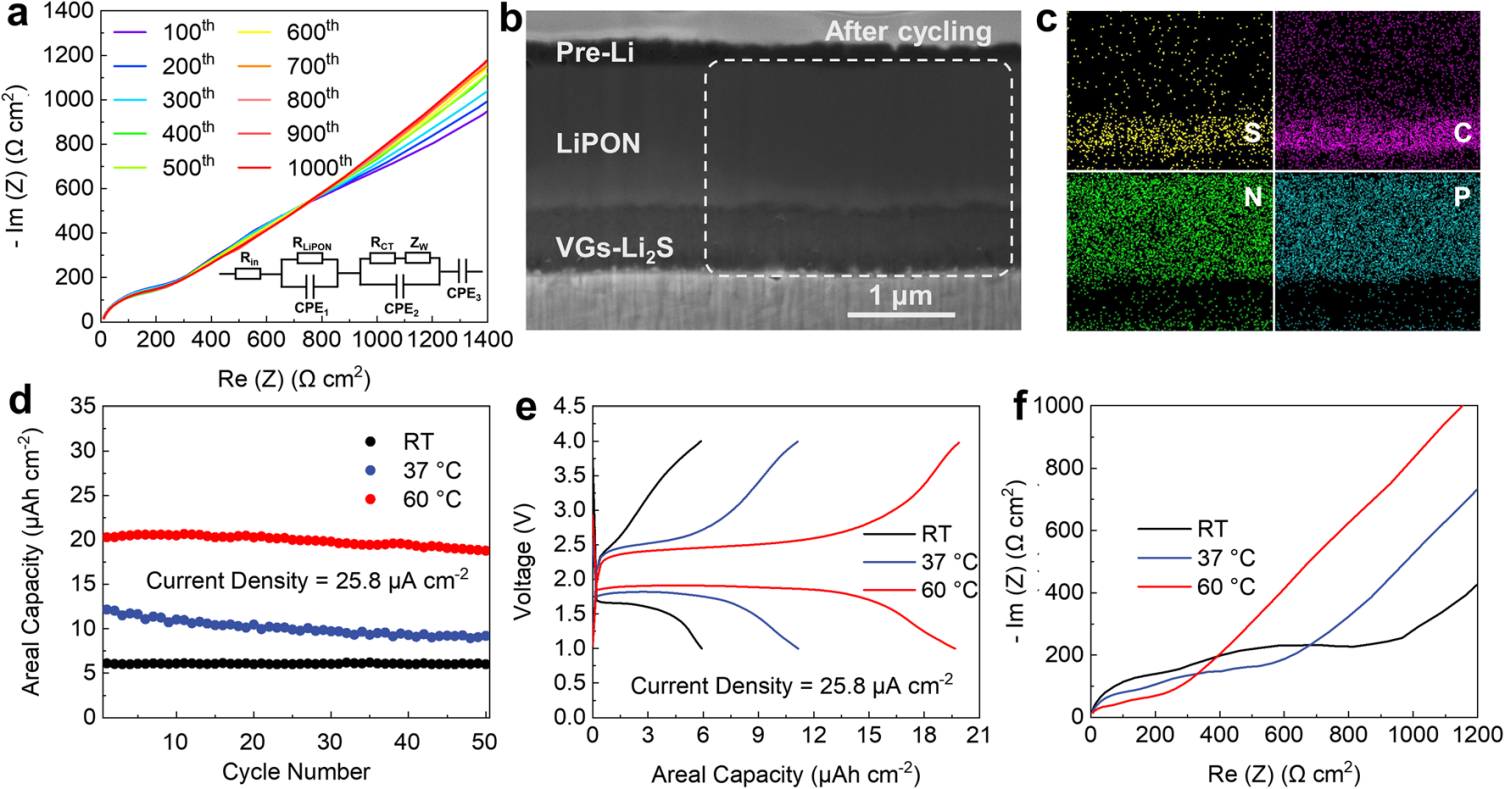
**a** Nyquist plots of the VGs-Li_2_S/LiPON/Pre-Li cell at different cycles. **b** FIB-SEM images of the VGs-Li_2_S/LiPON/Pre-Li cell after cycling, and **c** EDS mapping for elements of S, C, N and P. **d** Cycling performances of VGs-Li_2_S thin-film cathode at different working temperatures. **e** Voltage profiles, and **f** EIS at different working temperatures

EIS measurements were conducted to access the charge transport properties and Li^+^ diffusion behavior of the VGs-Li_2_S/LiPON/Pre-Li cell for every 100 cycles. The Nyquist plots and corresponding equivalent circuit are shown in Fig. 4a, where R_LiPON_ is the resistance of bulk LiPON and R_ct_ refers to the charge transfer resistance of VGs-Li_2_S/LiPON interface. The interfacial resistance of the Li/LiPON interface is not included in the equivalent circuit as it is negligible as compared to that of the VGs-Li_2_S/LiPON interface [6, 64]. The fitted EIS result (Fig. S9) reveal that *R*_ct_ is around 412 Ω cm^2^ after 1,000 cycles, suggesting tolerable interfacial resistance and intimate solid–solid contact between LiPON thin-film and VGs-Li_2_S cathode [6, 9]. The EIS curves remain almost the same during the whole cycling, evidencing that the unchanged charge transport properties and stable chemical/electrochemical properties of the cell [64, 65]. The diffusion coefficient of Li^+^ can be estimated from the Warburg impedance data and the corresponding fitting lines and computational methods part are presented in Fig. S10 [66]. The resultant diffusion coefficients of Li^+^ are 3.04 × 10^−20^, 2.79 × 10^−20^ and 2.63 × 10^−20^ cm^2^ s^−1^ after 200, 500 and 1,000 cycles, respectively. It is noteworthy that the diffusion coefficients of Li^+^ in thin-film cathode are relatively stable along with the cycling.

Furthermore, FIB-SEM combined with energy-dispersive X-ray spectrometry (EDS) element mapping were performed to further elucidate interfacial stability after cycling. Figure 4b of the cell after cycling exhibits well-defined interfaces both in the cathode and anode side, without any delamination, cracks or additional interface layer, demonstrating exceptional stability even after such long cycles. Despite the side reaction may occur between Li and LiPON to form Li_3_PO_4_, Li_3_N and Li_2_O et al., it will not affect the interfacial stability for long-term cycling because the side reaction is unabiding and the reaction products are beneficial to stabilize the Li metal anode [28, 49, 67, 68]. EDS elemental mapping results of S, C, N and P were captured at the region indicated by the white dashed line in Fig. 4b and presented in Fig. 4c to further verify the phase distribution of VGs-Li_2_S and LiPON. It shows a clear interface for the distribution of these elements, which are similar with the results before cycling (Fig. S11), indicating that cross-diffusion or intermixing among such elements did not occur significantly. These results elucidated that the LiPON solid electrolyte is favorably compatible with the Li anode and Li_2_S-based thin-film cathode.

The electrochemical properties of the VGs-Li_2_S/LiPON/Pre-Li cells at different working temperatures (25, 37, and 60 °C) were conducted to evaluate the potentially viable application scenarios, such as biological implantation (37 ℃) and extreme environment (60 °C). Figure 4d shows the gradually improving capacities with the elevated temperatures. When the temperature rises from RT to body temperature (37 °C), the capacity increases from 6 to 12.16 μAh cm^−2^. At 37 °C, the capacity begins to slowly decline at the very start but still maintains 9.16 μAh cm^−2^ after 50 cycles. Raising the temperature to 60°C rapidly rises the cell capacity to 20.47 μAh cm^−2^. Figure 4e shows the charge/discharge curves at different working temperatures. As depicted in curves, elevated temperatures make the plateaus prolonged and polarization narrowed, suggesting the capacity activation and the accelerated reaction kinetics of electrodes. To better understand the improved electrode kinetics, EIS measurements were performed. As shown in Fig. 4f, the impedance of bulk LiPON and the charge transfer process both decrease with increasing temperature. These demonstrated that the cell could adapt well to the changes in temperature and there are no side reactions occurred. A higher temperature contributes to not only increase the ionic conductivity of the LiPON, but also improve the energy conversion efficiency and energy density of the solid-state system.

### Electrochemical Performance of TFLSBs

**Fig. 5:**
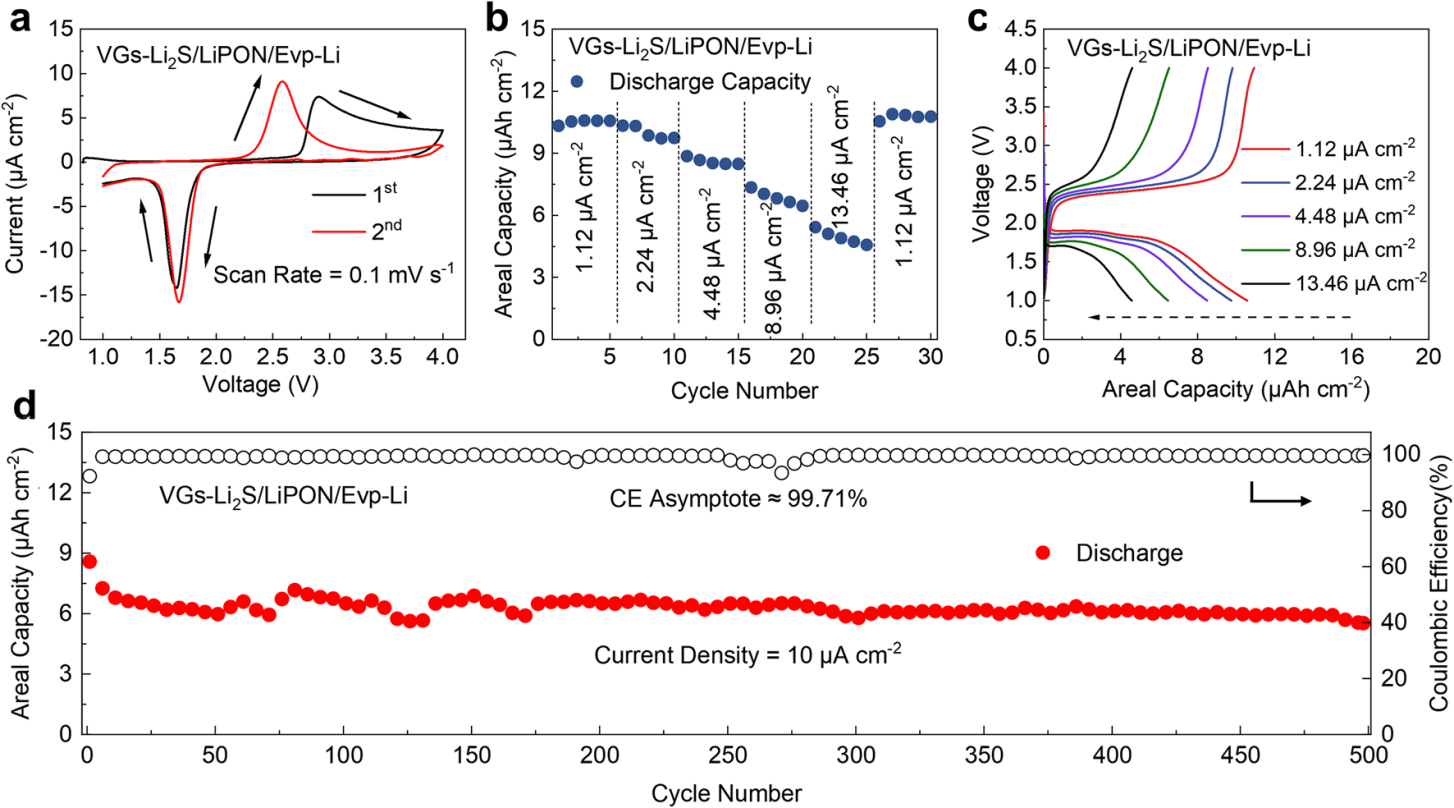
Electrochemical performances of the VGs-Li_2_S/LiPON/Evp-Li cell. **a** CV curves, **b** rate performance, **c** corresponding voltage profiles under different current densities, and **d** cycling performances at 10 μA cm^−2^

To further confirm that VGs-Li_2_S composite thin-film cathode material enables integration into TFLSBs, a thin-film cell architecture of VGs-Li_2_S/LiPON/Evp-Li is fabricated to evaluate its practical performance. Figure 5a displays the CV curve of the VGs-Li_2_S-based TFLSBs at a scan rate of 0.1 mV s^−1^. The higher oxidation peak at the first charge cycle is similar to that of the above model system analyses. Beyond that, only one couple of reduction and oxidation peaks appear corresponding to the direct conversion between S_8_ and Li_2_S. The charge/discharge profiles in the voltage window range of 1–4 V at 1.12 μA cm^−2^ are shown in Fig. S12. Notably, the voltage profiles of full cells still display similar behaviors, with the first charge plateau at 2.7 V and subsequent charge/discharge characteristic plateaus at 2.5 and 1.8 V, respectively. During the initial three discharge cycles, the discharge areal capacities of VGs-Li_2_S-based TFLSB were increased from 7.85 to 8.88 μAh cm^−2^, corresponding to the activation process of Li_2_S.

To better understand the redox behavior during the charge/discharge process, TFLSBs with Li thin-film anode was measured at different current densities (Fig. 5b). After an initial areal capacity of approximately 10.33 μAh cm^−2^ at 1.12 μA cm^−2^, the retention capacity reached a final value of 10.56 μAh cm^−2^ after 5 cycles. When cycling at 2.24, 4.48, 8.96 and 13.46 μA cm^−2^, the capacities remained at 10.36, 8.88, 7.35 and 5.41 μAh cm^−2^, respectively. Finally, the capacity returned to 10.88 μAh cm^−2^ at 1.12 μA cm^−2^, indicating that the VGs-Li_2_S/LiPON/Evp-Li cell exhibits excellent rate performance. Moreover, the typical charge/discharge profiles of the TFLSB at various current densities (Fig. 5c) also demonstrated apparent charge/discharge plateaus even at a high current density of 13.46 μA cm^−2^. As expected, the long-term cycling performance of VGs-Li_2_S-based TFLSBs were obtained at a current density of 10 μA cm^−2^ after CV test, which is presented in Fig. 5d. They deliver a discharge capacity of 5.52 μAh cm^−2^ after 500 cycles and reach an asymptotic CE value of 99.71%. The FIB-SEM image in Fig. S13 displays that the thin-film architecture of each layer in TFLSB is well preserved, suggesting that the Li_2_S-based thin film is well-compatible with the layer-by-layer stacking structure. Briefly, these results indicate that the VGs-Li_2_S thin-film cathode exhibits great promise for use in advanced TFBs.

The obtained results clearly reveal the feasibility of developing TFLSBs with superior stability, despite the extremely insulating nature of S-based material. The success of the VGs-Li_2_S-based TFLSBs with high performance could be ascribed to the following merits: (1) The electrodeposited Li_2_S exhibits high melting point and the tolerance of high vacuum, enabling the stability during the sputtering process of LiPON; (2) The three-dimensional conductive and porous VGs provide excellent conductivity and void space for highly insulated Li_2_S, facilitating the electron transport and accommodating the volume changes during the cycling; (3) The developed VGs-Li_2_S composite thin-film with a relatively flat surface ensures the intimate contact with LiPON, lowering the interfacial resistance; (4) The good interface stability between the VGs-Li_2_S and LiPON thin-film guarantees the stability of thin-film architecture during the long-term cycling processes. A deeper understanding of the lithium storage mechanism and structure-performance relationship of the developed TFLSBs can be gained through theoretical calculations and in-situ experiments in the future [18, 19, 25, 69, 70].

## Conclusions

In this study, the VGs-Li_2_S composite thin-film cathode was adopted to realize the fabrication of TFLSBs, which provides the grander prospect of transferring Li-S systems from the liquid into thin-film solid-state battery fields. Followed by LiPON solid electrolyte (≈ 1.5 μm) and Li thin-film anode deposition, we constructed VGs-Li_2_S/LiPON/Evp-Li TFLSBs and demonstrated their feasibility for the first time. Remarkably, the VGs-Li_2_S thin-film cathode could maintain superior stability for 3,000 cycles in the solid-state system, which can be attributed to the favorable compatibility of Li_2_S-based cathode with thin-film solid electrolyte and the capability of completely inhibiting polysulfide shuttle behavior. Meanwhile, the cell shows exceptional high temperature tolerance. Reversible cycling for 50 cycles at 60 ℃ was realized by VGs-Li_2_S/LiPON/Pre-Li systems, showing an improved areal capacity of 20.47 μAh cm^−2^. More importantly, encouraged by such superior performance, a VGs-Li_2_S-based TFLSB is fabricated as a practical consideration for assessing performance, which could stably deliver a discharge capacity of 5.52 μAh cm^−2^ after 500 cycles with a CE of 99.71%. In short, our work sheds light on designing high-performance TFLSBs and opens a new way for developing next-generation high-energy-density TFBs.

### Supplementary Information

Below is the link to the electronic supplementary material.

Supplementary file1 (PDF 708 kb)
