# Supplementary material for: All-Solid-State Thin-Film Lithium-Sulfur Batteries
Source: Nanomicro Lett. 2023 Mar 27;15:73. doi: 10.1007/s40820-023-01064-y (PMC10043110; doi:10.1007/s40820-023-01064-y)
Supplement: Supplementary file 1 — Supplementary file1 (PDF 708 kb) [file 40820_2023_1064_MOESM1_ESM.pdf]

Supporting Information for

All-Solid-State Thin-Film Lithium-Sulfur Batteries

Renming Deng[1,#], Bingyuan Ke[1,#], Yonghui Xie[1], Shoulin Cheng[1], Congcong Zhang[1], Hong Zhang[1,2,3], Bingan Lu[4,*], Xinghui Wang[1,2,3,*]

[1] College of Physics and Information Engineering, Institute of Micro-Nano Devices and Solar Cells, Fuzhou University, Fuzhou, 350108, P. R. China

[2] Fujian Science & Technology Innovation Laboratory for Optoelectronic Information of China, Fuzhou, Fujian 350108, P. R. China

[3] Jiangsu Collaborative Innovation Center of Photovoltaic Science and Engineering, Changzhou, 213000, P. R. China

[4] School of Physics and Electronics, Hunan University, Changsha, 410082, P. R. China

[#] Renming Deng and Bingyuan Ke contributed equally to this work.

* Corresponding authors. E-mail: luba2012@hnu.edu.cn (B. Lu); seaphy23@fzu.edu.cn (X. Wang)

Supplementary Figures

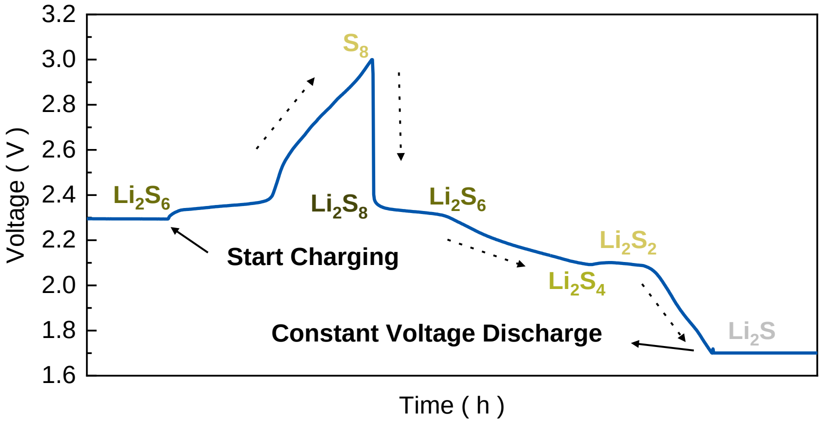

Fig. S1 Potential profiles of liquid-electrolyte lithium-sulfur battery using Li_2S_6 electrolyte

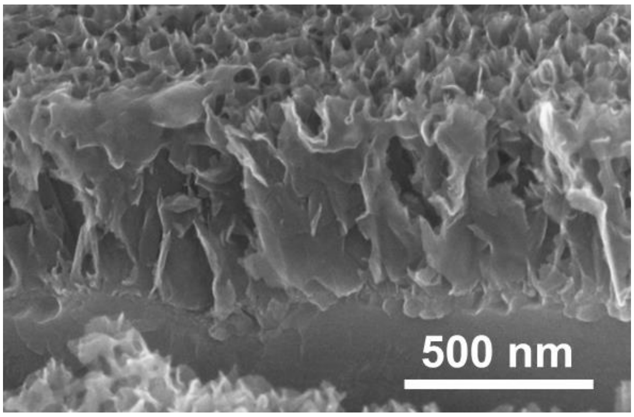

Fig. S2 Cross-section SEM image of the VGs

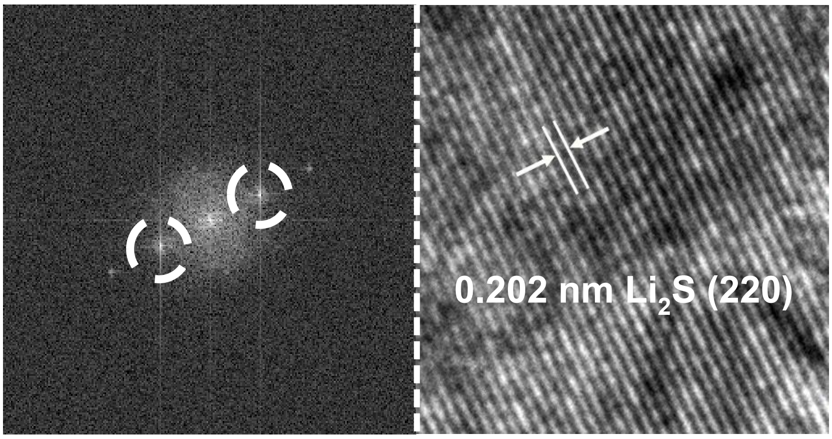

Fig. S3 FFT patterns, inverse FFT patterns

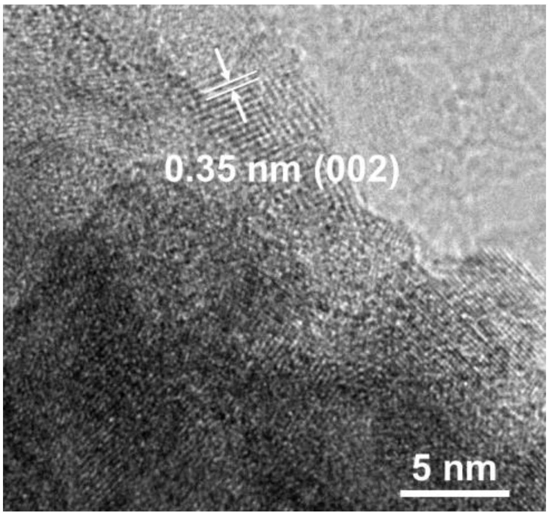

Fig. S4 HRTEM image of the graphene sheet.

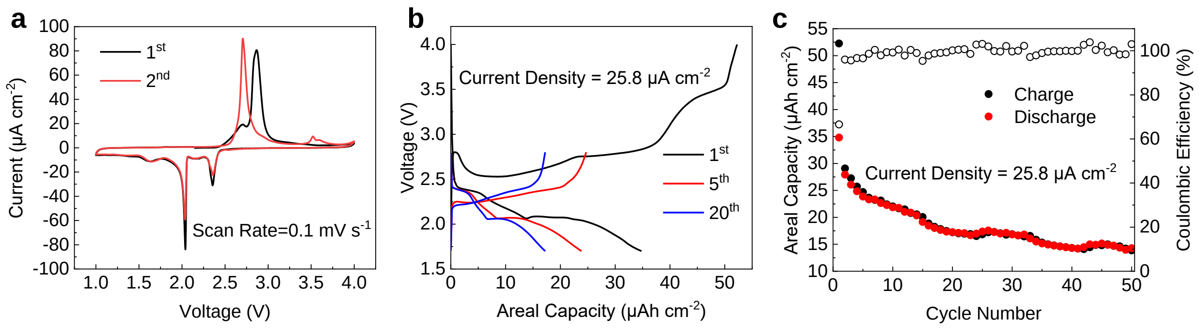

Fig. S5 Electrochemical performance of the VGs-Li_2S thin-film cathode in liquid system. **a** CV curves; **b** voltage profiles at a current density of 25.8 μA cm^{-2}; **c** cycling performances

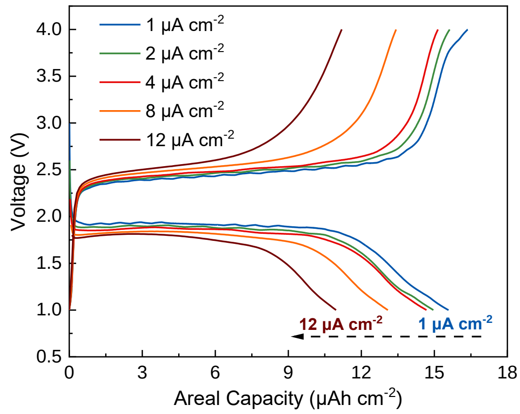

Fig. S6 Voltage profiles of the VGs-Li_2S thin-film cathode under different current densities

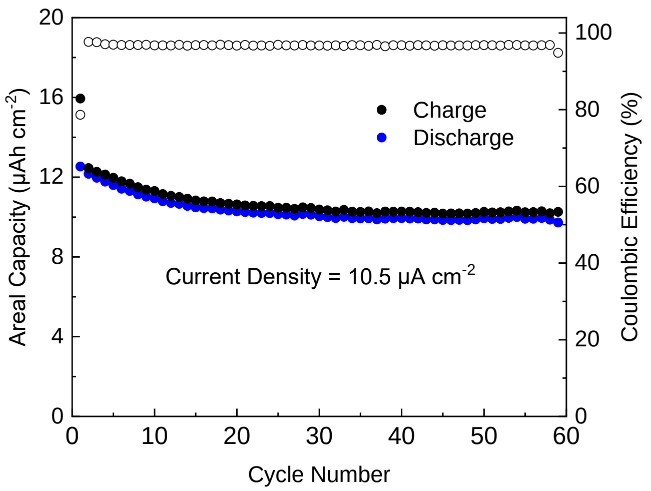

Fig. S7 Cycling performances of VGs-Li_2S/LiPON/Pre-Li cells at 10.5 μA cm^{-2} with an initial Coulombic Efficiency of 78.6%

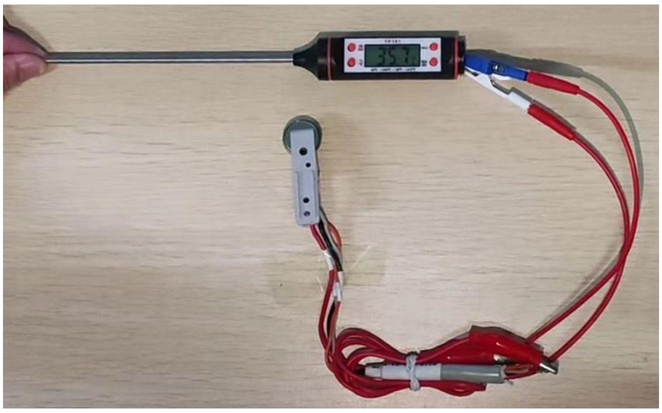

Fig. S8 Digital photos of the VGs-Li_2S/LiPON/Pre-Li cell (sealed with CR2025-type coin cell) connected with a small thermometer

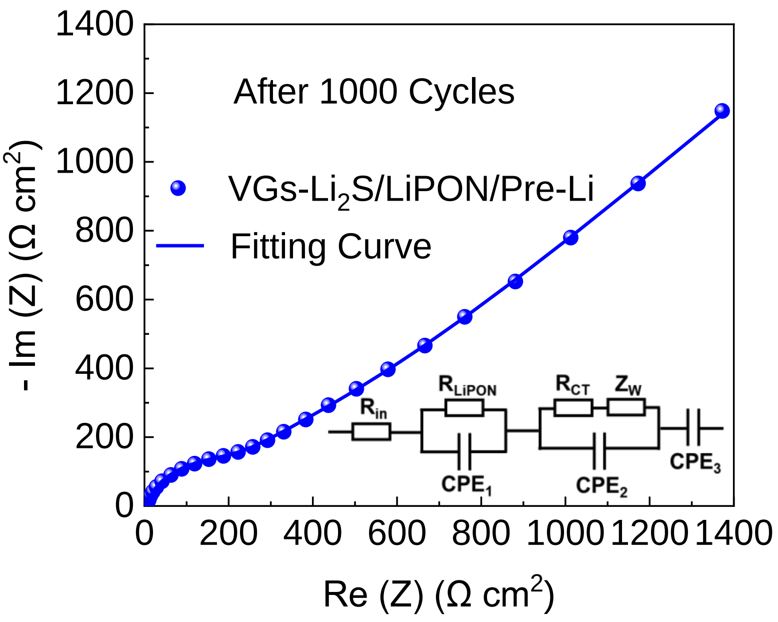

Fig. S9 The fitted Nyquist plots of the VGs-Li_2S/LiPON/Pre-Li cell after 1000 cycles

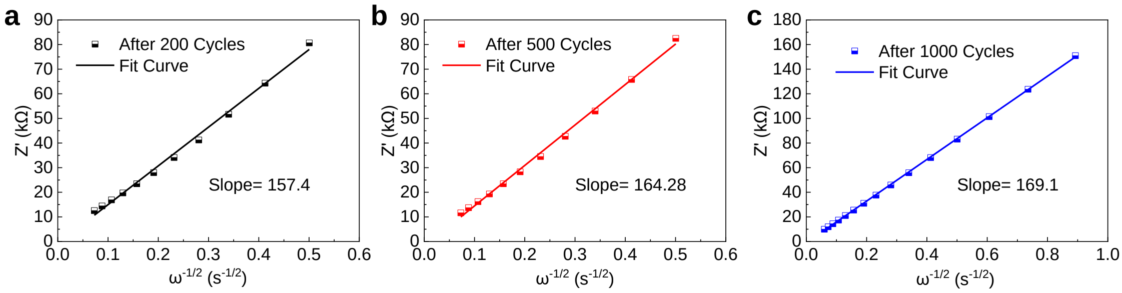

Fig. S10 The corresponding relationship between Z_{Re} and $\omega^{-1/2}$ in the low frequency region of after (**a**) 200, (**b**) 500 and (**c**) 1000 cycles

The Li^+ diffusion coefficient has been calculated from plots in the low frequency region using the following equation:

$$D = R^2T^2/2A^2F^4C^2\sigma_W{}^2 \qquad (S1)$$

Where R is the gas constant, T is the absolute temperature, A is the area of the cathode thin film, F is the Faraday constant, C is the concentration of Li^+, and σ_W is the Warburg impedance coefficient, which can be obtained from the slope of the real part of resistance (Z_{Re}) and the inverse square root of angular frequency ($\omega^{-1/2}$).

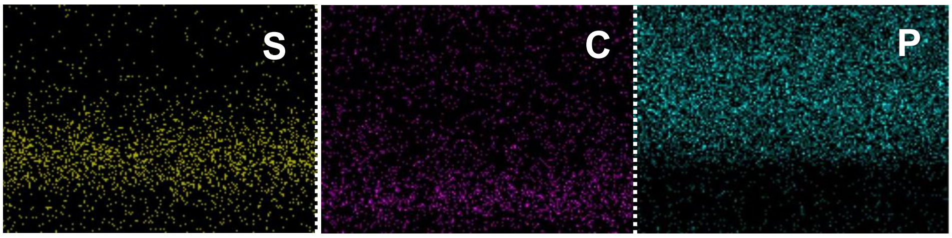

Fig. S11 EDS elemental mapping of S, C, and P at the VGs-Li_2S/LiPON interface before cycling

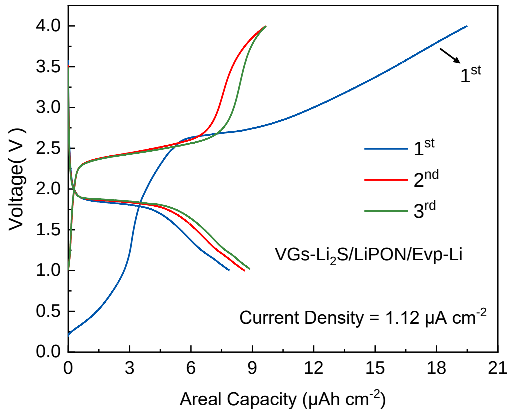

Fig. S12 Voltage profiles of the VGs-Li_2S/LiPON/Evp-Li cell at the first three cycles under a current density of 1.12 μA cm^{-2}

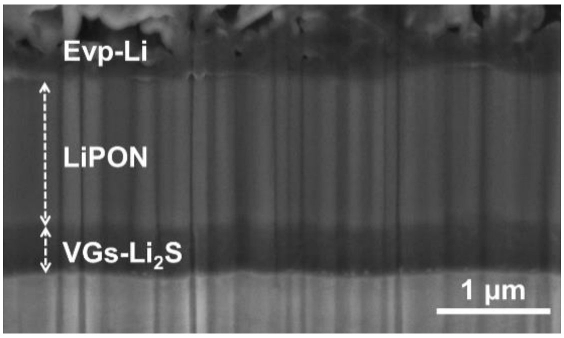

Fig. S13 FIB-SEM image of VGs-Li_2S/LiPON/Evp-Li cell after cycling
